# Improvement of sexual function in POEMS syndrome after combination therapy of Lenalidomide and dexamethasone

**DOI:** 10.1186/s13023-016-0461-8

**Published:** 2016-06-18

**Authors:** Hongbo Yang, Xufei Huang, Qianqian Cai, Chen Wang, Xinxin Cao, Daobin Zhou, Jian Li

**Affiliations:** Department of Endocrinology, Peking Union Medical College Hospital, Chinese Academy of Medical Sciences and Peking Union Medical College, Beijing, People’s Republic of China; Department of Hematology, Peking Union Medical College Hospital, Chinese Academy of Medical Sciences and Peking Union Medical College, Beijing, People’s Republic of China

**Keywords:** POEMS syndrome, Lenalidomide, Sexual function, Hypogonadism

## Abstract

**Background:**

POEMS syndrome is a rare paraneoplastic syndrome due to an underlying plasma cell neoplasm. Hypogonadism is the most common endocrine abnormality in POEMS syndrome. There is no data about improvement of hypogonadism and sexual dysfunction after appropriate treatment of POEMS syndrome so far. In this single-center prospective study, the efficacy of low-dose lenalidomide and dexamethasone combination therapy in the improvement of sexual and gonadal function in POEMS syndrome was evaluated.

**Results:**

Thirty-five patients with newly diagnosed POEMS syndrome were treated with Lenalidomide (10 mg daily for 21 days) and dexamethasone (40 mg once per week) for 12 cycles. The international index of erectile function (IIEF) in male patients, the female sexual function index (FSFI) in female patients, total testosterone, estradiol and vascular endothelial growth factor (VEGF) levels were reviewed. Sexual dysfunction was reported in 88.8 % (24/27)male and 90.9 % (10/11) female patients. 62.9 % (17/27) male and 25.0 % (3/12) female patients had hypogonadism. After 12-month treatment, the mean total FSFI score had increased from 17.1 ± 7.2 to 23.7 ± 7.2(*p* < 0.05). The arousal, orgasm and sex pain domains had been improved significantly. The mean IIEF scores had increased from 12.9 ± 13.0 to 20.5 ± 18.4 (*p* < 0.05). Erectile function, sexual desire and intercourse satisfaction had improved significantly at the end of treatment. No association between VEGF levels and sexual function improvement was found in both genders.

**Conclusion:**

Combination therapy with low-dose lenalidomide plus dexamethasone is successful in improving sexual function in POEMS syndrome.

## Background

POEMS (polyneuropathy, organomegaly, endocrinopathy, M-spike, skin changes) syndrome is a rare paraneoplastic syndrome characterized with multisystem involvement and markedly increased vascular endothelial growth factor (VEGF) levels [1]. VEGF is a growth factor for endothelial cells that induces vascular permeability, which is important in angiogenesis and often decreases with successful treatment [2].

Endocrinopathy is a central but poorly understood feature of POEMS. In Mayo Clinic series, about 84 % of patients had a recognized endocrinopathy, with hypogonadism as the most common endocrine abnormality, followed by thyroid dysfunction, abnormal glucose metabolism, and lastly by adrenal insufficiency [3]. In their report, 79 % male patients had subnormal total testosterone levels and 61 % reported erectile dysfunction. Gonadal dysfunction is a serious problem that reduces patients’ quality of life and interpersonal relationships. Most of the information regarding hypogonadism in POEMS syndrome is from isolated case reports or small series that have not systematically studied. Little is known about improvement of hypogonadism after appropriate treatment of POEMS syndrome.

Lenalidomide is a derivative of thalidomide, with powerful activity against malignant plasma cells as well as acting to decrease the levels of proinflammatory and proangiogenic cytokines. Recently, it has been showed that lenalidomide might play a promising role in the treatment of POEMS syndrome [4, 5]. In our previous work, the efficacy and safety of low dose lenalidomide plus dexamethasone had been demonstrated in patients with relapsed or refractory POEMS syndrome [2]. Here we conducted a pilot study to assess the efficacy of lenalidomide plus dexamethasone in the improvement of gonadal and sexual function of POMES syndrome in our center.

## Methods

### Patients

Forty-one consecutive patients with newly diagnosed POEMS syndrome (male 28, female 13) were treated with combination of low-dose lenalidomide and dexamethasone at Peking Union Medical College Hospital from April 2014 to November 2015. All patients met the diagnostic criteria defined by Dispenzieri, with two mandatory criteria (polyneuropathy and monoclonoal plasma cell proliferating disorder), at least one major criterion (sclerotic bone lesion, Castleman disease or VEGF elevation) and one minor criterion (organomegaly, edema, endocrinology, skin change, papillary edema or thrombocytosis) [6]. Excluded from the study were patients with a history of medication use, such as antidepressants, psychotropic drugs, beta-blockers and spirolactone, which can interfere with sexual function. No hormone replacement therapy was employed in patients with hypogonadism.

### Regimen and doses

All patients received lenalidomie (Revlimid; Celgene Corporation, Summit, NJ, USA) at a dose of 10 mg daily for 21 days of a 28-day cycle, plus oral dexamethasone at 40 mg once per week. Aspirin at a dose of 100 mg daily was prescribed for thromboprophylaxis.

### Study objectives

#### Assessment of sexual function

International Index of Erectile Function (IIEF) questionnaire and Female Sexual Function Index (FSFI) questionnaire were administered in male and female patients respectively at every visit. IIEF is sensitive and specific for detecting treatment-related changes in patients with erectile dysfunction [7]. In this 15-item self-reporting questionnaire, five separate domains of sexual function are measured, including erectile function, orgasmic function, sexual desire, intercourse satisfaction and overall satisfaction. FSFI is a 19-item multidimensional self-reporting measure, which quantifies six domains of female sexual dysfunction, including desire, arousal, lubrication, orgasm, satisfaction and pain. It has become the *de facto* “gold standard” in the assessment of female sexual function and an indispensable tool in clinical research of female sex dysfunction [8]. The reliability and validity of Chinese version of FSFI had been demonstrated in Chinese women [9].

#### Laboratory procedures

All blood samples were collected between 7 and 11 AM. Total testosterone in male and estradiol in female patients were measured with Access testosterone reagent and Access Estradiol reagent (Beckman Instrument, Inc. Chaska, MN, USA) in the central laboratory of Peking Union Medical College Hospital. Serum VEGF levels were measured with human Quantikine ELISA Kit (R&D Systems, Minneaplis, MN, USA). Normal serum VEGF was less than 600 pg/ml, which was determined in our previous work [10].

### Statistical analysis

Data analysis was performed with the statistical software package SPSS 22.0 (SPSS, Inc., Chicago, IL, USA). t-test was used to detect the statistical significance of the Hormone levels, VEGF levels, FSFI and IIEF scores before and after lenalidomide treatment. Spearman correlation test was performed to determine the association between IIEF/FSFI scores and hormone levels or serum VEGF. A value of *P* < 0.05 was considered statistically different.

## Results

### Baseline characteristics of participants

A total of 41 patients were enrolled in this study (male 28, female 13). The median age at diagnosis was 49 years (range, 21–70). One female patient and one male patient were ruled out because of sexually inactivity. Two female patients died during follow up and two male patients retreated. All other 35 patients finished the 12-months study. All the participants were married. Before treatment, the mean total FSFI score was 17.1 ± 7.2, much lower than that of normal Chinese female (27.3 ± 2.79) [9]. 90.9 % (10/11) female patients had FSFI score lower than 26.55, the cut-off value for a diagnosis of FSD [8]. 25.0 % (3/12) female patients had estradiol levels below the lower limit of normal range, including one patient with primary hypogonadism and two patients with secondary hypogonadism. Based on the score of IIEF, 88.8 %(24/27)male patients reported erectile dysfunction, including 66.7 %(18/27) with severe ED, 18.5 % (5/27) with moderate ED, 3.7 % (1/27) with mild to moderate ED. 62.9 % (17/27) male patients had total testosterone levels below the lower limit of normal range, including 6 patients with primary hypogonadism and 11 patients with secondary hypogonadism. The median VEGF level was 5155 pg/ml (range, 534–14328) before treatment. 94.7 % (38/39) had elevated VEGF than the cut-off value (600 pg/ml).

### Improvement of female sexual function after 12-months treatment

**Fig. 1:**
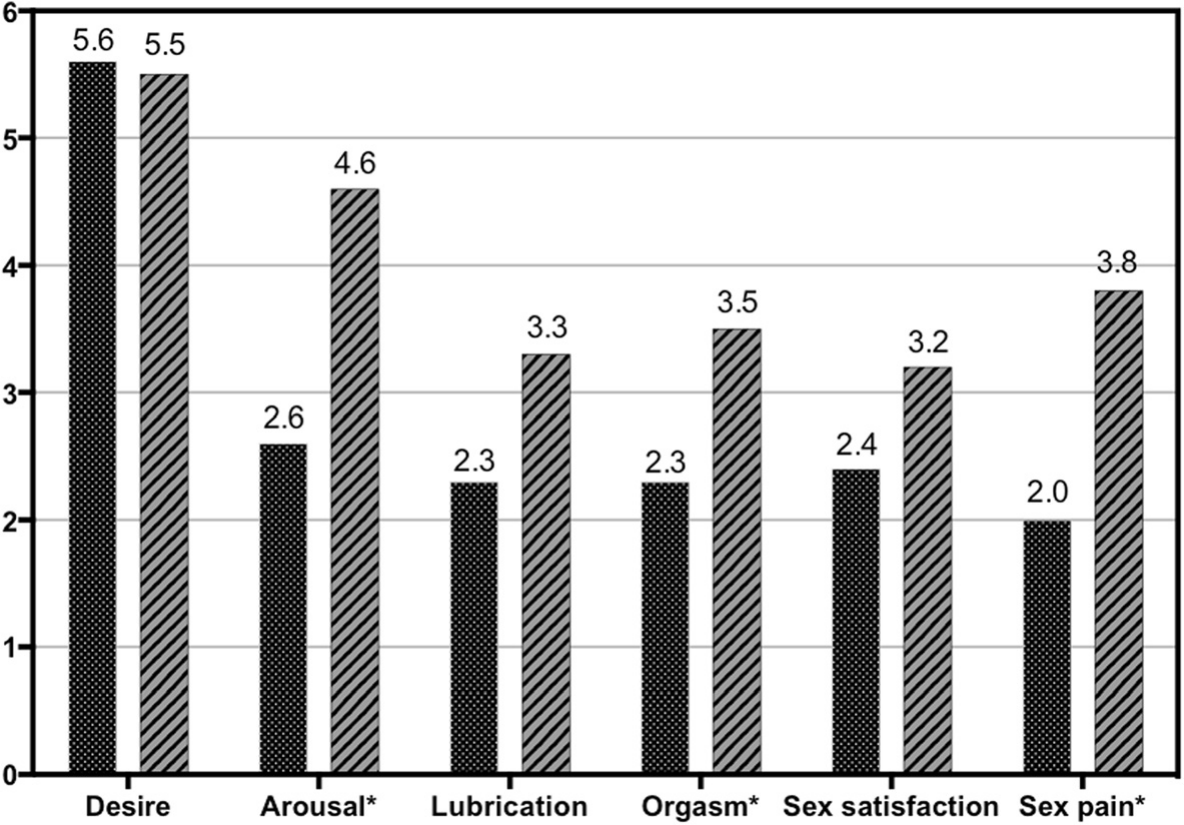
Mean FSFI distribution by domain before and after 12-month combination therapy. FSFI: Female Sexual Function Index. * *p* < 0.05

The mean total FSFI score had been improved to 23.7 ± 7.2 after 12-month treatment. On the basis of the total FSFI score, FSD was diagnosed in 10 of 11 patients (90.9 %) at baseline and 7 of 10 patients (70.0 %) after treatment. The arousal, orgasm and sex pain domains had been improved significantly at the end of 12-month treatment (Fig. 1 and Table 1). Changes in mean scores from baseline to the end of the study for individual questions assessing desire, lubrication and sex satisfaction were not statistically different.

**Table 1:** Changes in FSFI scores and laboratory parameters after 12-month combination therapy in Female patients

	Baseline (*N* = 12)	End of study (*N* = 10)	95 % confidence interval of the diff (*N* = 10)		*P* value
			Lower	Upper	
FSFI Domains Desire	5.6 ± 0.9	5.5 ± 1.0	−0.9	0.6	0.468
Arousal	2.6 ± 1.9	4.6 ± 1.8	0.6	4.0	0.002*
Lubrication	2.3 ± 1.9	3.3 ± 2.0	−0.8	3.3	0.084
Orgasm	2.3 ± 1.9	3.5 ± 2.1	−0.6	3.4	0.050*
Satisfaction	2.4 ± 1.2	3.2 ± 2.0	−0.6	2.1	0.112
Pain	2.0 ± 1.6	3.8 ± 2.4	−0.7	4.8	0.046*
Total FSFI score	17.1 ± 7.2	23.7 ± 7.2	1.5	13.6	0.003*
FSH (IU/L)	12.38 ± 13.47	33.20 ± 28.75	4.45	61.95	0.067
LH (IU/L)	8.05 ± 9.09	17.26 ± 18.81	−1.55	36.07	0.017*
E_2_ (pg/mL)	81.92 ± 62.62	59.50 ± 92.79	−93.99	113.69	0.771
VEGF (pg/mL)	6190.5 ± 3979.3	1468.2 ± 1495.4	908.7	8871.7	0.004*

*SD* standard deviation

### Association between female sexual function, estradiol and VEGF levels

In female patients, levels of estradiol were 81.92 ± 62.62 pg/ml at baseline and 59.50 ± 92.79 pg/ml at the end of study (*p* = 0.771). There was no significant improvement of estradiol levels. The mean VEGF levels decreased from 6190.5 ± 3979.3 pg/ml at baseline to 1468.2 ± 1495.4 pg/ml at the end of treatment (*p* = 0.004). No association between VEGF levels and estradiol levels was found (*p* = 0.580, Spearman test). Nor did the association between VEGF levels and FSFI scores (*p* = 0.841, Spearman test).

### Improvement of male sexual function after 12-months treatment

**Fig. 2:**
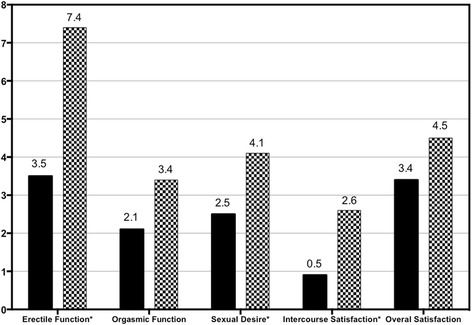
Mean IIEF distribution by domain before and after 12-month combination therapy * *p* < 0.05

After 12-month treatment, the mean IIEF scores have increased from 12.9 ± 13.0 to 20.5 ± 18.4 (*p* < 0.05). Patients with severe ED have decreased from 66.7 % (18/27) to 40.0 % (10/25). Ratio of patients with normal erectile function have increased from 11.1 % (3/27) to 36.0 (9/25) (Table 2). Erectile function, sexual desire and intercourse satisfaction had improved significantly at the end of treatment (Fig. 2, Table 3). Changes in mean scores from baseline to the end of the study for individual questions assessing orgasmic function and overall satisfaction were not statistically different.

**Table 2:** Severity of erectile dysfunction at baseline and at the end of study

	Baseline (*N* = 27)	End of study (*n* = 25)
Severity of ED (EFD score),n%		
Severe (<11)	18(66.7)	10(40.0)
Moderate (11 ~ 16)	5(18.5)	5(20.0)
Mild to moderate (17 ~ 21)	1(3.7)	1(4.0)
Mild (22 ~ 25)	0(0.0)	0(0.0)
Normal (>25)	3(11.1)	9(36.0)

**Table 3:** Changes in IIEF scores and laboratory parameters after 12-month combination therapy in male patients

	Baseline (*N* = 27)	End of study (*N* = 25)	95 % confidence interval of the diff (*N* = 25)		*P* value
			Lower	Upper	
IIEF Domains					
Erectile Function	3.6 ± 5.4	7.3 ± 8.9	−3.3	10.6	0.012*
Orgasmic Function	2.2 ± 2.5	3.0 ± 3.2	−1.7	3.4	0.106
Sexual Desire	2.6 ± 1.4	3.8 ± 2.6	−1.1	3.6	0.012*
Intercourse Satisfaction	0.9 ± 2.8	2.2 ± 3.6	−0.4	3.9	0.022*
Overall Satisfaction	3.6 ± 2.1	4.2 ± 2.6	−2.1	3.4	0.228
Total IIEF Score	12.9 ± 13.0	20.5 ± 18.4	−6.1	21.4	0.009*
FSH (IU/L)	10.77 ± 12.51	19.32 ± 12.50	6.82	31.82	0.411
LH (IU/L)	8.32 ± 5.07	10.15 ± 9.45	0.70	19.60	<0.001*
Total T (nmol/L)	5.38 ± 3.23	11.30 ± 5.67	−1.54	2.78	<0.001*
VEGF (pg/mL)	5965.0 ± 2860.3	1355.6 ± 1563.4	1422.4	7862.0	<0.001*

### Association between male sexual function, total testosterone and VEGF levels

In male patients, levels of total testosterone were 5.38 ± 3.23 nmol/L at baseline and had been improved to 11.30 ± 5.67 nmol/L at the end of study (*p* < 0.001). The mean VEGF levels decreased from 5965.0 ± 2860.3 pg/ml at baseline to 1355.6 ± 1563.4 pg/ml at the end of treatment. No association between total testosterone levels and IIEF scores was found. There was no association between VEGF levels and IIEF scores or testosterone levels, neither.

## Discussion

The present study explored the under-investigated area of sexual quality of life in patients with POEMS syndrome. Our results demonstrated that low-dose lenalidomide plus dexamethasone combination therapy is successful in improving sexual function in patients with POEMS syndrome.

Hypogonadism is the most common endocrine disorders in POEMS syndrome. Because of the low incidence, there were only case reports about hypogonadism in POEMS so far [11, 12]. In the Mayo series, 79 % (26/38) male patients had hypogonadism and 61 % (23/39) reported erectile dysfunction [3]. In our reported series, hypogonadism was seen in 52.2 % (26/46) male patients. Impotence (89 %) and gynecomastia (12 %) were common findings in male patients [13]. Sasano et al. reported the first case that showed an improvement in gonadotropin secretion after corticosteroid therapy [14]. After three months of corticosteroids treatment, the 49-year-old male patient showed an improvement in danadotropin secretion but no considerable change in the secretion of the other hormones. Recently, Chu et al. reported their experience of a patient with POEMS syndrome with long-term therapy with lenalidomide. The total testosterone levels had increased from 55 ng/dl to 624 ng/dl after one year [15].

There are no clinical trials assessing improvement of hypogonadism or sexual function after appropriate treatment of POEMS syndrome. Our study is the first report about improvement of sexual function in POEMS syndrome. Sexual dysfunction had been reported in 90.9 % female patients and 88.8 % male patients at baseline. In male patients, the mean IIEF scores have increased from 12.9 ± 13.0 to 20.5 ± 18.4 at the end of study. Patients with severe ED have decreased from 65.4 % to 42.3 %. Ratio of patients with normal erectile function had increased from 11.5 % to 34.6 %. Erectile function, sexual desire and intercourse satisfaction had improved significantly at the end of treatment. In female patients, the mean total FSFI score had been improved to 23.7 ± 7.2 after 12-month treatment. In female patients, FSD was diagnosed in 90.9 % at baseline and decreased to 70.0 % at the end of study. The arousal, orgasm and sex pain domains had been improved significantly at the end of 12-month treatment.

Sexual function is affected by multiple factors such as anatomical, physiological, psychological and social factors, and in consequence, it can lead to decreased quality of life. Autonomic nerve function plays an important role in sexual function. It was reported that autonomic fibers might be preserved despite major involvement of the large motor fibers in POEMS patients [16]. Hypogonadism is another important factor contributing to sexual dysfunction. In our study, hypogonadism was found in 25.0 % female patients and 62.9 % male patients. At the end of study, there was no significant improvement of estradiol levels in female patients but a significant increase of total testosterone levels was found in male patients. Surprisingly, no association between total testosterone levels and IIEF scores was found.

The pathogenesis of endocrinopathy is not well understood. Circulating antibodies against hormones or specific hormone receptors have not been found. No specific structural changes had been found in endocrine glands [17]. VEGF is a growth factor for endothelial cells that induces an increase in vascular permeability, is important in angiogenesis, and often decreases with successful therapy. There were accumulating evidences suggesting that the high level of VEGF contributed to some specific features of POEMS syndrome, such as extravascular volume overload, organomegaly, hemangioma, and peripapillary retinal thickness [18, 19]. In this study, after 12-month treatment, the mean VEGF levels decreased significantly in both gender. But no association between VEGF levels and IIEF score or FSFI score changes had been demonstrated. There are several other options for treatment of POEMS syndrome, including melphalan, thalidomide and transplantation. All these regimens are effective in decreasing VEGF levels. Whether the effects on sexual and gonadal function are specific to lenalidomide/dexamethasone or not is another interesting issue to investigate.

The present study had several limitations. The main limitation of this study is the number of participants. Taking into account the fact that POEMS syndrome is a rare disorder, small populations do not guarantee a very strong insight. A larger number of patients will provide more information between sexual function and gonadal hormone level changes especially in female patients. We also did not collect data regarding educational status or social economic status.

## Conclusion

In summary, low-dose lenalidomide plus dexamethasone combination therapy is successful in improving gonadal function in patients with POEMS syndrome.

## Abbreviations

FSFI, female sexual function index FSFI is a 19-item multidimensional self-reporting measure, which quantifies six domains of female sexual dysfunction, including desire, arousal, lubrication, orgasm, satisfaction and pain. It has become the *de facto* “gold standard” in the assessment of female sexual function and an indispensable tool in clinical research of female sex dysfunction; IIEF, international index of erectile function IIEF is a 15-item self-reporting questionnaire, in which five separate domains of sexual function are measured, including erectile function, orgasmic function, sexual desire, intercourse satisfaction and overall satisfaction. IIEF is sensitive and specific for detecting treatment-related changes in patients with erectile dysfunction; POEMS syndrome, POEMS (polyneuropathy, organomegaly, endocrinopathy, M-spike, skin changes) syndrome is a rare paraneoplastic syndrome characterized with multisystem involvement; VEGF, vascular endothelial growth factor VEGF is a growth factor for endothelial cells that induces vascular permeability
